# Helios but not CD226, TIGIT and Foxp3 is a Potential Marker for CD4^+^ Treg Cells in Patients with Rheumatoid Arthritis

**DOI:** 10.33594/000000080

**Published:** 2019

**Authors:** Mengru Yang, Yan Liu, Biyao Mo, Youqiu Xue, Congxiu Ye, Yutong Jiang, Xuan Bi, Meng Liu, Yunting Wu, Julie Wang, Nancy Olsen, Yunfeng Pan, Song Guo Zheng

**Affiliations:** a Division of Rheumatology, Department of Internal Medicine, the Third Affiliated Hospital, Sun Yat-Sen University, Guangzhou, China,; b Center for Clinical Immunology, the Third Affiliated Hospital, Sun Yat-Sen University, Guangzhou, China,; c Division of Rheumatology, Department of Internal Medicine, Hainan General Hospital, Haikou, China,; d Division of Rheumatology, Department of Internal Medicine, Guangdong Second Provincial Central Hospital, Guangzhou, China,; e Division of Rheumatology, Department of Medicine, Penn State University Hershey College of Medicine, Hershey, PA, USA,; f Department of Internal Medicine, Ohio State University College of Medicine and Wexner Medical Center, Columbus, OH, USA

**Keywords:** Rheumatoid arthritis, Regulatory T cells, Helios, CD226, TIGIT

## Abstract

**Background/Aims:**

Rheumatoid arthritis (RA) is a progressive, chronic, even disabling systemic autoimmune disease. Imbalance between pathogenic immune cells and immunosuppressive cells is associated with the pathogenesis and development of RA and other autoimmune diseases. As Foxp3 is also expressed on activated CD4^+^ cells in the presence of inflammation, the identification of Treg cells in patients with RA remains a challenge.

**Methods:**

Comprehensive analyses were carried out by Flow cytometry. Expression of Helios, CD226, T cell immunoreceptor with Ig and ITIM domains clinical samples and healthy controls.

**Results:**

We have systemically examined three potential markers, Helios, CD226 and TIGIT, that are possibly related to Treg identification, and found that Helios expression on CD4^+^Foxp3^+^ cells was decreased and negatively correlated with the disease activity of RA patients, while CD226 and TIGIT both showed elevated expression levels in CD4^+^Foxp3^+^ cells in RA patients and they were not associated with disease activity of RA patients.

**Conclusion:**

Taken together, our findings indicate that CD4^+^CD25^hi^CD127^low/-^Foxp3^+^Helios^+^ may represent the real Treg cell population in patients with RA.

## Introduction

Rheumatoid arthritis (RA) is a chronic inflammatory systemic autoimmune disease, which has an incidence of 0.28% in China [1]. The pathogenesis of this disease is incompletely understood, but includes genetic factors, environmental stress, disordered immunity, dysregulated cytokines and stromal cell activation [2]. Immune intolerance to auto-antigens is essential to the development of RA [3]. Recently, besides biologics, other therapeutics such as intracellular signal inhibitors and cellular therapies have progressively attracted attention of scientists in RA treating [4–7]. Treg cells immunotherapy is emerging as one important therapeutic strategy for development.

The subset of regulatory T cells (Treg) within the CD4^+^CD25^+^Foxp3^+^ T cell population plays a critical role in maintaining immunological self-tolerance [8–11]. Studies have shown that the reduction in the numbers and/or function of Treg and phenotypic defects would lead to autoimmune intolerance and abnormal immune responses to auto-antigens which could lead to various autoimmune diseases [12–15], including RA, systemic lupus erythematosus, ankylosing spondylitis, ANCA-related vasculitis and other diseases. In RA patients, Tregs can be divided into at least two parts: one part is present in the peripheral blood while the other is present in inflammatory sites such as synovial tissue [16]. Our study is aimed at investigating peripheral blood Tregs.

Although CD4^+^CD25^high^ and CD127^low/−^ cell populations were traditionally used to identify the human Treg subset and Foxp3 is an important and even unique marker for Treg cells [17–19], this is not case for the identification of Treg cells in patients with autoimmune diseases since some activated non-Treg cells also express Foxp3, CD25 and have also lost CD127 [20]. Recently, researchers have found some additional molecules that are associated with Treg cell identification, for example, CTLA-4, GITR, ICOS, CD39, Nrp-1 and so on [21]. In addition to these, three other molecules, Helios, CD226 and TIGIT have been reported [22–25]. Nonetheless, most studies have been conducted in healthy subjects.

Helios, a member of the Ikaros transcription factor family, is a nuclear factor expressed in early development of T cells. Previous study found that Helios may play a significant role in controlling the stability and function of Treg [26]. Researchers found that the Foxp3^+^Treg cell subset in peripheral blood and synovial fluid of patients with rheumatic diseases (RA and spondyloarthritis) was associated with Helios expression [27]. Interestingly, Helios gene expression may have critical roles in the pathogenesis of RA *via* its effect on Foxp3 gene epigenetic modification [28]. Additionally, as a subset of Tregs, Helios^+^Foxp3^+^ Tregs are expanded in active SLE [29]. Whether Helios expression in Tregs is associated with the pathogenesis of RA remains to be determined. We therefore aimed to analyze Helios expression in Tregs from RA patients and attempted to find an association with disease activity.

CD226, also known as DNAM-1, is a leukocyte differentiation antigen that is mainly expressed on CD4^+^ and CD8^+^ T cells, monocytes and NK cells. CD226 was also identified as a co-stimulatory receptor that shares its ligands, poliovirus receptor (PVR, CD155) and Nectin-2 (PVRL2, CD112), with the co-inhibitory receptor TIGIT [30, 31]. A previous study suggested that the expression of CD226 might affect the immunosuppressive effect of Tregs [31]. In addition, a large number of studies have confirmed that the CD226 gene is associated with a variety of autoimmune diseases including RA, systemic lupus erythematosus, juvenile idiopathic arthritis, and others [32–35]. Accordingly, we aimed to explore the association between CD226 and Tregs from RA patients.

T cell immunoreceptor with Ig and ITIM domains (TIGIT), a co-inhibitory molecule, can inhibit T cell activation and proliferation [36–38]. A previous study found that TIGIT was increasingly expressed in healthy human nTreg which may be involved in stability and inhibition functions of Treg [31]. The previously-mentioned study implied that elevated TIGIT levels in RA synovial fluid might inhibit abnormal immune responses in RA patients [39]. However, the latest study reported that TIGIT showed higher expression in RA patients, in peripheral blood CD3^+^CD4^+^ T cells and CD3^+^CD8^+^ T cells [40]. This finding implies that TIGIT may have other effects on the pathogenesis of RA, in addition to acting as a negative co-stimulatory molecule.

In the current study, we have systemically investigated a cohort of patients with RA in China to determine the ability of these molecules to identify Treg subsets and have also evaluated their correlation with disease activity and therapy.

## Materials and Methods

### Human subjects

Peripheral blood samples (4 ml) were obtained from 51 healthy volunteers and 74 patients with RA who met the 1987 American Rheumatism Association criteria or the 2010 ACR/ EULAR Classification criteria for RA. In addition, 150 ml of peripheral blood was collected from other 4 RA patients for suppression assays. All human studies have been approved by the Research Ethical Committee of the Third Affiliated Hospital at Sun Yat-sen University. Before study, written informed consents were received from all participants.

Patients were divided into different groups using the following: (1) the DAS28 score (high disease activity > 5.1, moderate disease activity < 5.1 and > 3.2, low disease activity < 3.2 and >2.6, remission (inactive disease activity) < 2.6); (2) whether the patient was receiving any treatments in the past 3 months; (3) whether the patient has been treated in the past 3 months with any DMARDs (Methotrexate, Sulfasalazine, Hydroxychloroquine, Leflunomide, Tripterygium glycosides and Total Glucosides of Paeony Capsules), Steroids or TNF-α inhibitors (TNFi, including Tocilizumab, Etanercept and Infliximab). The characteristics of the patients and healthy controls are shown in Table 1.

### Flow cytometry

Freshly obtained peripheral blood mononuclear cell (PBMC) were stained with anti-CD4 (-FITC from Biolegend, OKT4, USA), anti-CD25 (-PE from Biolegend, BC96, USA), anti-CD127 (-PECY7 from Biolegend, A019D5, USA), anti-CD226 (-APC from Biolegend, 11A8, USA), anti-TIGIT (-APC from eBioscience, MBSA43, USA). Intracellular detection of Foxp3 with anti-Foxp3 (-Percp-Cy5.5 from eBioscience, 236A/E7, USA) and Helios with anti-Helios (-APC from Biolegend, 22F6, USA) was performed on fixed and permeabilized cells *via* Foxp3 Staining Buffer Set (eBioscience, USA). Cell fluorescence was acquired on BD LSR Fortessa (BD Biosciences, USA) and analyzed with FlowJo software (version 7.6.5; Tree Star). We usually acquired 10, 000 events in FSC. CD4-FITC positive and SSC gates were used to delineate CD4^+^ cells, then gated with CD25-PE and CD127-PECY7 in these cells, and the acquisition gate was designed on the CD4^+^CD25^high^CD127^low/−^ cells.

### Suppression assays

We extracted PBMC from 150 ml peripheral blood of RA patients, we then separated and purified these cells to obtain purified lymphocytes by nylon wool column. These cells were stained with anti-CD4, anti-CD25, anti-CD127, and anti-TIGIT. The CD4^+^CD25^high^CD127^low/−^ TIGITT cell, CD4^+^CD25^high^CD127^low/−^ TIGIT^−^ T cell and CD25- T cells were sorted by fluorescence-activated cell sorting (FACS) analysis using BD InFlux (BD Biosciences, USA). The cell purity of sorted cells was more than >99%. Freshly sorted CD25^−^ T cells were labeled with 1 uM carboxyfluorescein succinimidyl ester (CFSE; Biolegend, USA) for 15 minutes at 37.8°C as T_eff_ cells. CD4^+^CD25^high^CD127^low/−^ TIGIT^+^ T cell and CD4^+^CD25^high^CD127^low/−^ TIGIT^−^ T cell were sorted as Treg cells. Then TIGIT^−^ or TIGIT^+^ Treg were cocultured with T_eff_ cells at different ratios in culture medium which containing 30ng/mL soluble anti-CD3 monoclonal antibodies (mAb) (OKT-3; Miltenyi biotec, Germany) and irradiated APC (30 Gy, 1:1 ratio) in 96- well U-bottomed culture plates for 72 hours. Then we stained these cells with anti-CD8a (-APC from Biolegend, USA). Lastly, we tested the proliferation of CD8^+^ T cells to compare the suppressive function between TIGIT^+^ Treg and TIGIT^−^ Treg by FACS. We gated on CD8^+^ cells from T effector cells to display the suppressive effects of CD4^+^ Treg cells, since this excludes the interference of reading results from the contaminating CD4^+^ cell population.

### Statistical analysis

Data analysis was performed using GraphPad Prism version 5.0 software (GraphPad Software). All data were expressed as mean values ± the standard error of the mean (S.E.M). Comparison between groups was analyzed by Mann-Whitney test. Correlation analyses were carried out using Spearman’s rank correlation test. *p*<0.05 was considered statistically significant.

## Results

### Frequencies of CD4^+^CD25^hi^CD127^low/−^ Treg in peripheral blood of RA patients and healthy controls

**Fig. 1.**
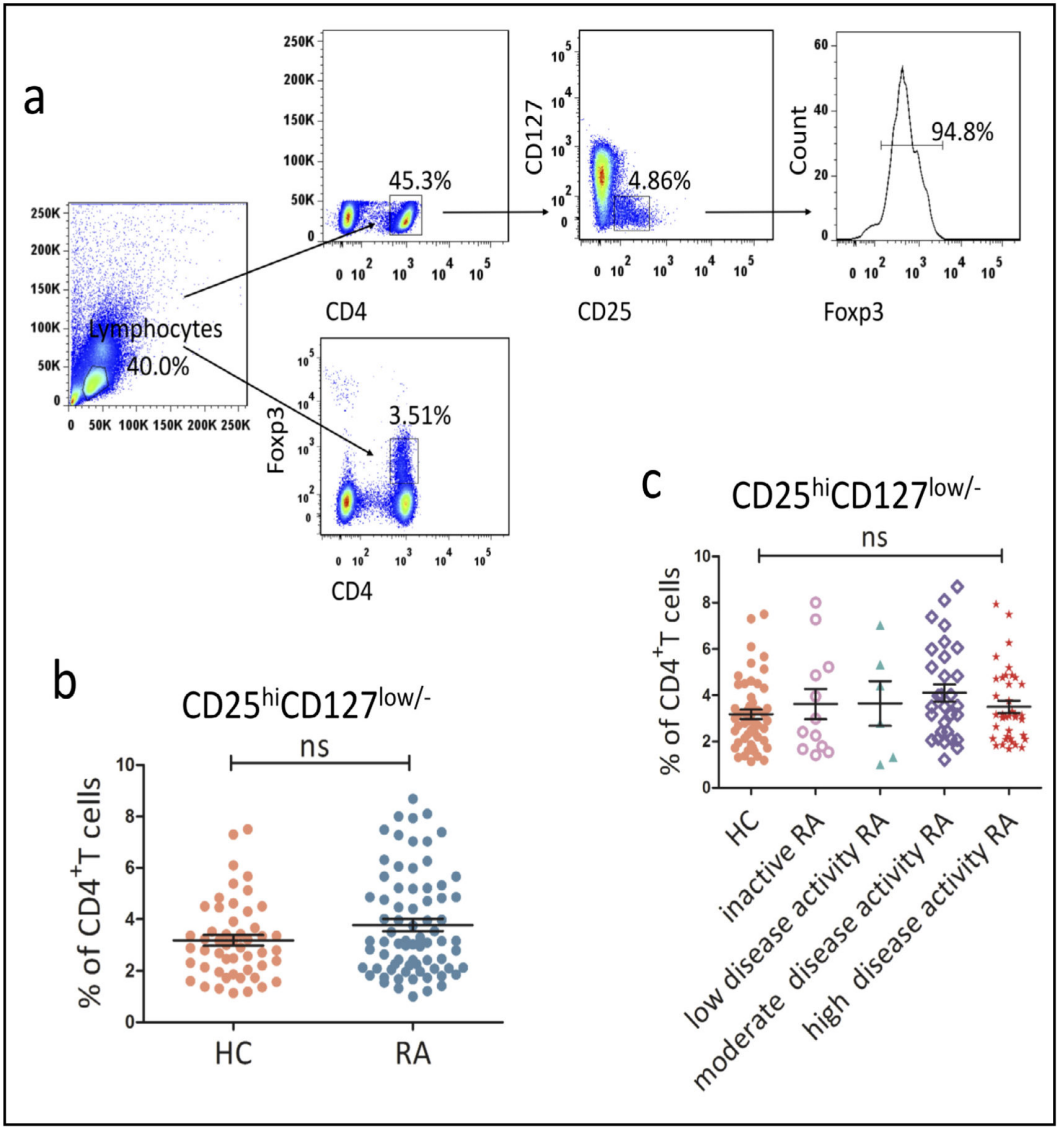
Frequencies of CD4^+^CD25^hi^CD127^low/−^ Treg in peripheral blood of RA patients and healthy controls. (a) CD25^+^CD127^low/−^ Treg cells were gated within the CD4^+^ T cell population in peripheral blood mononuclear cells (PBMCs) from RA patients. Almost all CD4^+^CD25^hi^CD127^low/−^ cells are Foxp3^+^ cells. (b) Frequencies of CD4^+^CD25^hi^CD127^low/−^ Tregs were compared between healthy controls (3.17±1.49%) and RA patients (3.77±1.98%) (*p*>0.05). (c) Frequencies of Tregs were compared between healthy controls and RA patients with different levels of disease activity (*p*>0.05). The p value was measured with Mann-Whitney test. n.s., not significant.

Given that CD4^+^CD25^hi^CD127^low/−^ best represents Treg cells in human, initial studies examined the frequency of these cells in RA patients and healthy controls. CD4^+^ cells were selected from the peripheral blood mononuclear cell by FACS and CD25^hi^ and CD127^low/−^ cells were further isolated for subsequent analyses as previously [41]. Almost all CD4^+^CD25^hi^CD127^low/−^ T cells in peripheral blood were positive for Foxp3 (Fig. 1a). We found no significant differences in the percentages of CD25^hi^CD127^low/−^ cells within CD4^+^ T cells between peripheral blood samples from RA patients (n=74) and those from healthy controls (n=51) (*p>0.05*) (Fig. 1b). Even though the contribution of age had a slight difference (shown in Fig. S1 - for all supplemental material see www.cellphysiolbiochem.com), it had no impact on the overall results. Similarly, we found no gender specific differences in Treg frequencies (shown in Fig. S2). Moreover, there were no meaningful differences in the frequencies of CD4^+^CD25^hi^CD127^low/−^ T cells in RA patients with different levels of disease activity (*p>0.05*) (Fig. 1c).

**Fig. 2.**
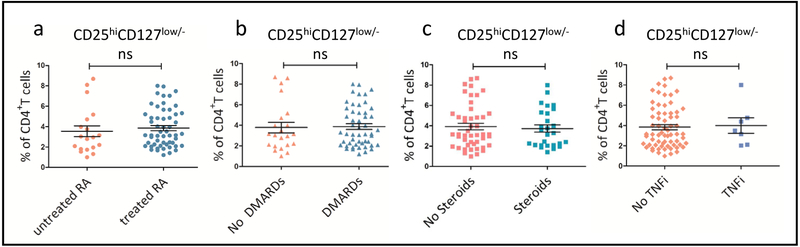
Frequencies of CD4^+^CD25^hi^CD127^low/−^ Tregs in peripheral blood of RA patients with different therapies. Frequencies of Tregs were compared according to whether RA patients were on treatment (a), such as DMARDs (b), Steroids (c) or TNF-α inhibitors (d) (*p*>0.05). The p value was measured with Mann-Whitney test. n.s., not significant.

Next, we grouped RA patients by their treatment received for at least the previous 3 months. This analysis showed no significant differences in CD4^+^CD25^hi^CD127^low/−^ T cell frequencies between treated RA and untreated RA *(p>0.05*) (Fig. 2a), including using DMARDs or no DMARDs (*p>0.05*) (Fig. 2b), using steroids or no steroids (*p>0.05*) (Fig. 2c), and using TNFi or no TNFi (*p>0.05*) (Fig. 2d).

### Expression of Helios in Treg and CD226, TIGIT on Treg in RA patients and their correlation with disease activities

**Fig. 3.**
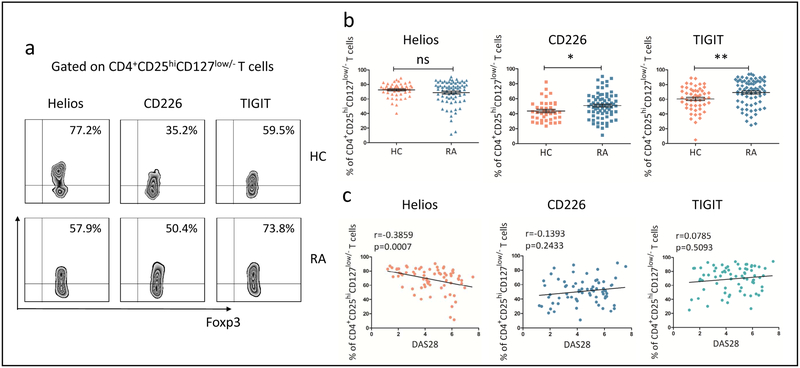
Expression of Helios in Tregs, CD226 and TIGIT on Tregs in RA patients and their correlation with disease activity, (a) Helios, CD226, TIGIT expression in or on peripheral blood Tregs was detected by using flow cytometry, (b) The expression levels of Helios in Tregs were compared between HCs (72.35±9.25%) and RA patients (68.69±16.463%) (*p*>0.05) (left); the expression levels of CD226 on Tregs were compared between HCs (43.54±13.70%) and RA patients (50.84±16.91%) (*p*<0.05) (middle); and the expression levels of TIGIT on Tregs were compared between HCs (60.44± 16.68%) and RA patients (69.23±17.75%) (*p*<0.01) (right). The p value was measured with Mann-Whitney test, n.s., not significant, * and ** indicate *p* <0.05 and *p* <0.01, respectively, (c) The expression of Helios in Tregs was negatively correlated with DAS28 score (r=−0.3859, *p=*0.0007) (left), and the expression of CD226 (middle) and TIGIT (right) were not associated with DAS28 score (*p*>0.05). Correlation analyses were carried out using Spearman’s rank correlation test.

To determine whether Treg cells marked by CD4^+^CD25^hi^CD127^low/−^ have an association with RA pathogenesis, development and therapy, we next investigated the expression of Helios in peripheral blood Treg, and CD226, TIGIT, on these cells by using flow cytometry (Fig. 3a). Results showed that the expression of Helios in CD4^+^CD25^hi^CD127^low/−^ T cells tended to be decreased in RA patients compared to healthy controls, but a significant difference was not reached *(p>0.05)*. At the same time, the expression of CD226 on CD4^+^CD25^hi^CD127^low/−^ T cells in RA patients was significantly elevated, as compared to healthy controls *(p<0.05)*. Similarly, the expression of TIGIT on Tregs in RA patients was also significantly elevated (*p<0.01)* (Fig. 3b).

We further analyzed the correlation between the DAS28 score of RA patients and the expression of the above-mentioned molecules. Data indicated that the expression of Helios was negatively correlated with the DAS28 score (r=−0.3859, *p*=0.0007, n=74). Meanwhile, the correlation between the expression of CD226 or TIGIT on Tregs and the DAS28 score in RA also was investigated, but no significant correlation was found (Fig. 3c).

### Expression of Helios in different disease activity of RA patients and with different treatments

**Fig. 4.**
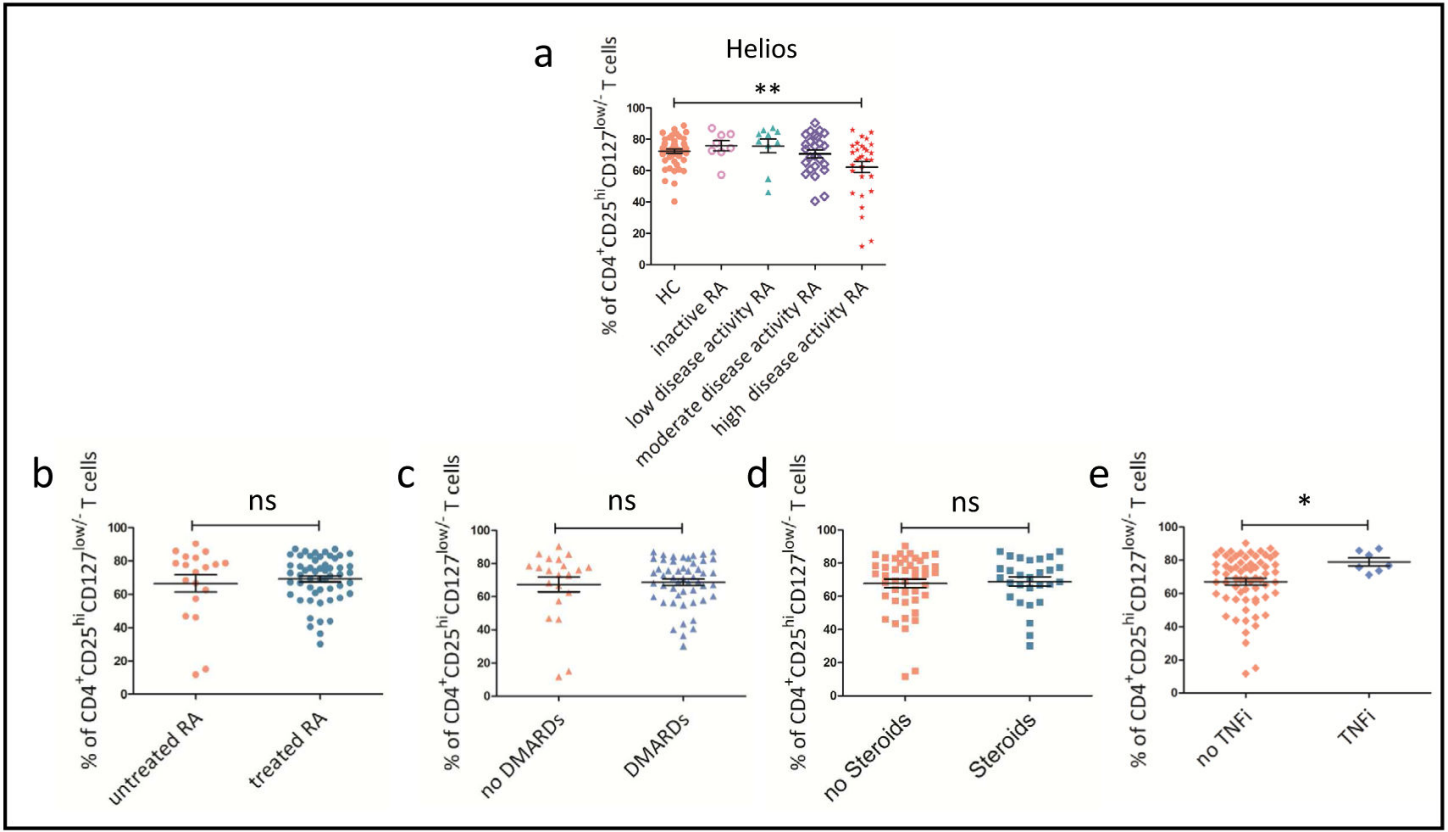
Expression of Helios in RA patient groups with different disease activity and with different treatments. (a) Helios was significantly lowerd in high disease activity RA than in healthy controls (p<0.01). (b)The expression of Helios in Tregs from RA patients was compared according to whether patients were on treatments such as DMARDs (c), Steroids (d) (*p*>0.05) or TNFi (e) (*p*<0.05). The p value was measured with Mann-Whitney test. n.s., not significant, * and ** indicate *p* <0.05 and *p* <0.01, respectively.

The aforementioned results demonstrated that there was no significant difference in Helios expression between RA patients and healthy controls. Interestingly, additional data demonstrated that Helios was significantly decreased in high disease activity RA patients compared to healthy controls (*p<0.01*) (Fig. 4a), suggesting that Helios may be associated with the immune process in patients with active disease.

On the other hand, in order to evaluate the influence of different treatments on Helios expression in RA Treg, we grouped RA patients by their treatments for at last 3 months (*p>0.05*) (Fig. 4b). Consequently, we found DMARDs and steroids have little effect on the Helios expression level (Fig. 4c, 4d) (*p>0.05*). However, treatment with TNFi was associated with a higher frequency of Helios^+^ Treg cells, suggesting that TNFi might contribute to the expression of Helios in CD4^+^CD25^hi^CD127^low/−^ T cells in RA patients (*p<0.05*) (Fig. 4e).

### Helios is much more specific than Foxp3 on CD4^+^CD25^hi^ T cells for RA patients with active disease

**Fig. 5.**
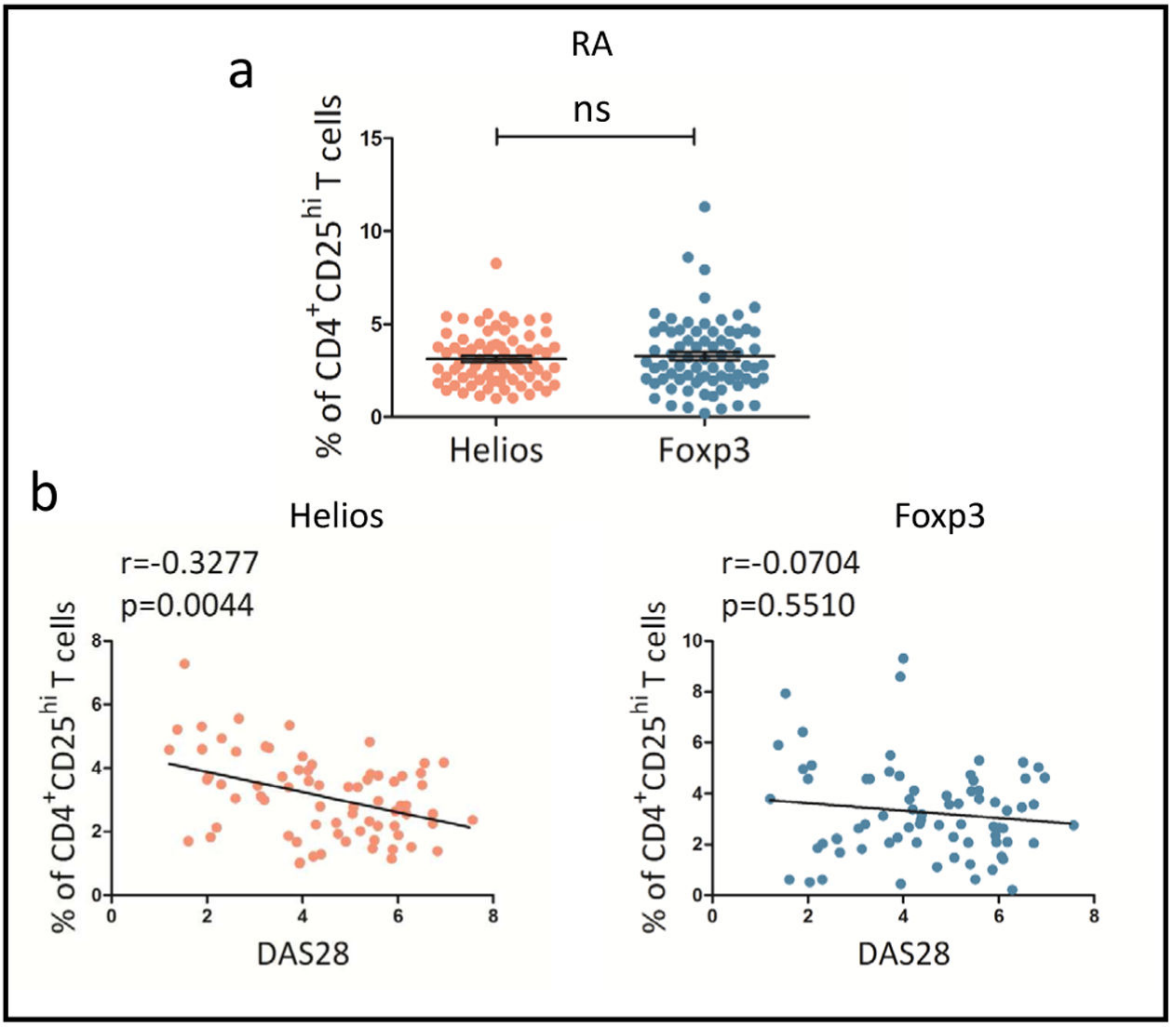
Helios is more closely associated with disease activity than Foxp3 on CD4^+^CD25^hi^ T cells in RA patients. (a) The expression of Helios in CD4^+^CD25^hi^ T cells was similar to Foxp3 on RA Tregs (*p*>0.05). (b) The expression of Helios in CD4^+^CD25^hi^ T cells was negatively correlated with DAS28 score (r=−0.3277, *p*=0.0044) (left), while the expression of Foxp3 was not associated with DAS28 score on these T cells (*p*>0.05) (right). The p value was measured with Mann-Whitney test. Correlation analyses were carried out using Spearman’s rank correlation test. n.s., not significant.

To analyze whether Helios is a more effective indicator of the degree of disease activity in patients with RA, we compared the expression of Helios and Foxp3 in CD4^+^CD25^hi^ T cells in RA patients. Data showed no significant difference in the expression of Helios and Foxp3 in Tregs of RA patients (p>0.05) (Fig. 5a). Next, we analyzed the correlation between the expression of Helios or Foxp3 in CD4^+^CD25^hi^ T cells and the DAS28 score in RA patients.

Interestingly, we observed that the expression of Helios was still negatively correlated with the DAS28 score (r=−0.3277, *p*=0.0044, n=74), conversely, the expression of Foxp3 in CD4^+^CD25^hi^ T cells did not correlate with the DAS28 score (Fig. 5b). These results suggested that the CD4^+^CD25^hi^ Helios^+^ T cells may be a better marker for the Treg cell population than others that are currently used, and the frequency of these Helios^+^ identified cells also may be informative regarding disease activities and treatment with TNFi in RA patients.

**Fig. 6.**
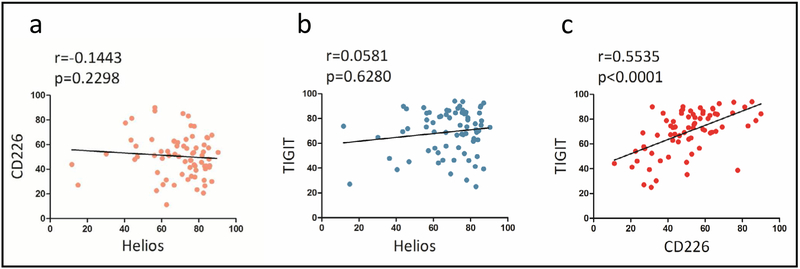
The correlation among different Treg related molecules in RA Tregs. (a) The expression of Helios in Tregs was not correlated with CD226 expression on Treg (*p*>0.05). (b) The expression of Helios in Tregs was not associated with TIGIT expression on Treg (*p*>0.05). (c) A linear association between CD226 expression and TIGIT expression was detected (r=0.5535, *p*<0.0001, n=74). Correlation analyses were carried out using Spearman’s rank correlation test.

We also sought to determine the correlation among these Treg relative molecules in RA Treg cells. We observed there was no significant correlation between Helios and CD226, as well as between Helios and TIGIT in RA Treg (Fig. 6a, b). Nonetheless, we found that the expression of CD226 was positively correlated with that of TIGIT on RA Treg cells (r=0.5535, *p*<0.0001, n=74). It is likely that both CD226 and TIGIT share similar ligands (Fig. 6c).

### TIGIT-expression Treg cells has no suppressive capacity

**Fig. 7.**
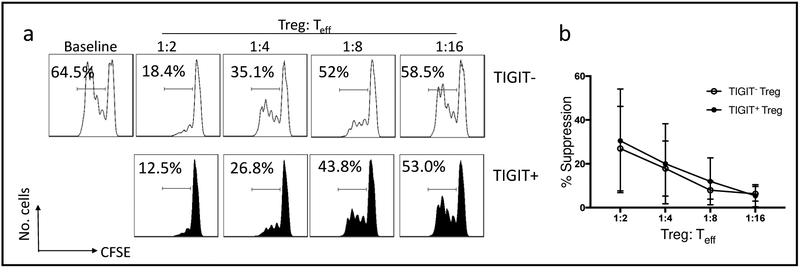
TIGIT-expression on Treg cells has no significant suppressive capacity. (a) Expanded human Treg subsets co-cultured with CFSE-labeled CD25^−^ T cell and OKT3 (30ng/ml) for 72 hours in the presence of irradiated APCs. The CFSE dilution was examined by flow cytometry and data is representative of 4 independent experiments. (b) Statistical summary of 4 RA patients. There were no significant differences in suppressive capacity between TIGIT^+^ Treg and TIGIT^−^ Treg subsets (*p*>0.05).

To prove that the above-mentioned TIGIT is not a distinct Treg marker in RA patients, we performed a functional assay on TIGIT-expressing Treg. Data showed that both CD4^+^CD25^hi^CD127^low/−^ TIGIT^+^ Treg and CD4^+^CD25^hi^CD127^low/−^ TIGIT^−^ Treg could inhibit CD25^−^ T cells in Treg / T_eff_ at the indicated ratios, but there were no significant differences in suppressive capacity of TIGIT^+^ Treg and TIGIT^−^ Treg (*p*>0.05) (Fig. 7).

## Discussion

Rheumatoid arthritis (RA) is a chronic autoimmune disease which is characterized by systemic inflammation, persistent synovitis, structural joint damage and bone destruction [5–7]. In theory, the reduction in the numbers and/ or function of Treg would lead to autoimmune intolerance and abnormal immune responses. The importance of Tregs in collagen-induced arthritis models has been well documented [42, 43].

In fact, there has been controversy over the frequency of Tregs in the peripheral blood of RA patients compared to healthy controls. Some studies reported that Tregs are reduced in the peripheral blood of patients with RA [14, 44–47]. Nonetheless, other studies also demonstrated the converse results, showing that the frequency of Treg cells is increased [48, 49]. In addition, some studies reported similar frequency of Treg cells between RA patients and healthy controls [50]. It is likely that different races, geographic regions, disease activities and treatments might have an impact on the frequency of Treg cells. To explain the observed results, some researchers suggested that there might be a negative feedback regulation system during Treg involvement in the immune response [40]. Others mentioned that the increase in the number of Tregs in RA may be related to the long-term use of DMARDs [45]. Another possible reason is that the phenotypic definition of Tregs was not uniform. CD25, CD127 and Foxp3 were usually used to identify the Treg cells population in most studies. However, Foxp3 expression is not unique for human Treg cells, since it is also expressed on other activated human cells [51–53].

In addition to CD25, CD127 and Foxp3, recent studies have identified several new molecular markers such as Helios, CD226 and TIGIT that identify Treg cells in healthy subjects [22, 54, 55]. To determine the role and significance of these molecules in identifying Treg cells in RA patients, we detected the expression of Helios, CD226 and TIGIT on CD4^+^CD25^hi^ and CD4^+^CD25^hi^CD127^low/−^ T cells in peripheral blood and correlated the frequency to ages, gender, disease activities, and treatments in patients with RA. We found Helios expression on CD4^+^CD25^hi^CD127^low/−^ T cells, unlike that of CD226 and TIGIT, tended to be decreased in RA patients, especially in those with high disease activity. Our results are different from another previous study [28]. In their investigation, the Helios mRNA expression was significantly higher in RA patients compared to healthy controls. This difference could be explained by the treatment intervention and analysis methodology. All patients in this previous study were untreated RA patients, whereas most of our patients have been treated with various therapies. Additionally, we have used the disease activity as an important parameter, and this was not considered in the other study. Moreover, our data showed Helios expression was negatively correlated with RA disease activity. Considering that Foxp3 is also an especially crucial marker involved in stability and suppressive capacity of Treg in healthy subjects, we next compared Foxp3 and Helios expression and their correlation with RA disease activity. It is interesting that Helios but not Foxp3 is significantly associated with RA disease activity. At the present time, we are not certain as to the exact role of Helios on Treg cells, although others have recently reported that expression levels of Helios affect the stability of Tregs.

A previous study found that tocilizumab but not TNF-α inhibitors or abatacept increased Helios expression in CD4^+^ T cells in its responders [23]. Interestingly, our data indicated that Helios has a higher level of expression in patients in the TNFi group. We propose several reasons that might explain this difference. First, different methods were used. While we used FACS to identify the Helios protein levels, this other study used DNA microarray analysis to identify Helios mRNA levels. Second, the subjects selected were different. We compared Helios expression in RA patients between TNFi treatment or none while this study compared the Helios mRNA on individual patients before and after the use of TNFi. Third, we focused on CD4^+^CD25^hi^CD127^low/−^ Foxp3^+^ T cells while this study gated on CD4^+^ T cells. Additionally, differences in race and in the dosage of TNFi may also be responsible for the different results. Larger samples and multi-center studies are needed to address this in the future.

It has been well recognized that TNF-α is critically involved in the pathogenesis of RA and many other autoimmune diseases, however, its effect on Treg biological activity has been controversial [56]. Our data now provide a new line of evidence that TNFi therapy may ameliorate the RA severity through Helios^+^ Foxp3^+^ Treg cells. It has been generally accepted that patients treated with TNFi have an increased percentage of Foxp3^+^Treg cells with restored regulatory function of these cells which are associated with the reduction of some pro-inflammatory cytokines like TNF-α and IL-6. In addition, a previous study has suggested Foxp3^+^ Treg in peripheral blood of RA patients was associated with Helios expression [28]. Therefore, we here proposal that TNFi therapy could also affect the expression and/or expansion of Helios by regulating a balance between immune regulation and inflammatory factors. Therefore, we plan future studies to further explore the underlying mechanisms by which TNF-α inhibitor treatment restores and increases the Helios^+^Foxp3^+^ Treg cells.

Collectively, we inferred that Helios may be a reliable marker for identifying Treg cells and play an important role in Treg immunosuppressive function in RA, especially in high disease activity patients, but the underlying mechanism remains to be explored. Additionally, we speculated Helios might be used as a clinical marker of RA disease activity in the future, although this would require further study. We believe this observation has high clinical relevance, since current studies have not yet ideally identified Treg cell population in patients with RA and other autoimmune diseases. As a co-stimulatory receptor, CD226 was previously shown to be involved in T cell activation, affecting the immunosuppressive capacity of Tregs [22, 30]. Our study has found that there was no association between CD226 and disease activity in RA, conversely, its frequency is even increased in RA patients. Therefore, whether CD226 is involved in RA through the regulation of T cells remains to be further explored. Meanwhile, as an inhibitory co-stimulatory molecule, TIGIT was also previously considered to be involved in stability and inhibitory function of Tregs [31, 57]. The present study is the first to investigate the expression of these molecules on CD4^+^CD25^hi^CD127^low/−^ Tregs from peripheral blood of RA patients and their correlation with disease activity. The elevated expression of TIGIT in RA synovial fluid may inhibit abnormal immune responses in RA patients, which suggests that TIGIT may have a therapeutic role in RA [39]. However, we found TIGIT also showed elevated expression on peripheral blood Treg in RA patients and there was no correlation between TIGIT and RA disease activity. These results seem to be controversial in the light of inhibitory characteristics of TIGIT. And our suppression assays also illustrated that TIGIT had no significant effect on suppressive capacity of Tregs. In fact, in recent studies, TIGIT was noted to be expressed in follicular T helper cells (Tfh) [58, 59]. Thus, its role in immune regulation is uncertain since Tfh cells participate in B cell immunity and antibody production in RA [60]. The role of TIGIT in the pathogenesis of RA is a subject that merits further exploration.

## Conclusion

To our knowledge, this is the first demonstration that Helios could be a better marker than Foxp3 to identify Treg cells in RA patients. Helios, but not CD226 and TIGIT, expression in CD4^+^CD25^hi^CD127^low/−^ T cells was negatively correlated with the disease activity of RA patients. Moreover, further studies including longitudinal analyses the frequency of CD4^+^CD25^hi^CD127^low/−^Helios^+^ Treg in RA patients starting TNF-α inhibitors would be of interest since this may eventually help to evaluate responses to TNF-α inhibitors therapies and prognosis in patients with RA. Furthermore, we plan also to determine whether Helios^+^ and Helios^−^ Treg subsets in autoimmunity have different epigenetic and other molecular profiles.

## Supplementary Material

Supplemental Figures 1+2

## Figures and Tables

**Table 1.** Characteristics of the patients with rheumatoid arthritis (RA) and healthy controls. Note: ESR: erythrocyte sedimentation rate; CRP: C-reactive protein; DAS28: 28-joint Disease Activity Score; RF: rheumatoid factor; anti-CCP: anti-cyclic citrullinated peptide; NSAIDs: Nonsteroidal anti-inflammatory drugs; DMARDs: Disease-modifying anti-rheumatic drugs; TNF-α inhibitor: tumor necrosis factor alpha inhibitor

Parameter	RA (N=74)	Healthy controls (N=51)
Sex, no. men/ no. women	16/58	20/31
Age, years, mean ± S.D.	46.50±15.46	42.08±11.48
ESR, mm/ hour, mean ± S.D.	49.74±33.29	-
CRP, mg/dl, mean ± S.D.	32.39±41.88	-
DAS28, mean ± S.D.	4.54±1.54	-
RF, no. positive/ no. negative	45/20	-
Anti-CCP, no. positive/ no. negative	37/15	-
Treatment no. of patients		-
None	18	
NSAIDs	18	
DMARDs	51	
TNF-α inhibitor	7	
Steroids	26	
