# Supplementary material for: Helios but not CD226, TIGIT and Foxp3 is a Potential Marker for CD4+ Treg Cells in Patients with Rheumatoid Arthritis
Source: Cell Physiol Biochem. Author manuscript; Available in PMC 2020 Jan 6. (PMC6943339; doi:10.33594/000000080)
Supplement: Supplemental Figures 1+2 [file NIHMS1065446-supplement-Supplemental_Figures_1_2.pdf]

Supplementary Material

Helios But Not CD226, TIGIT and Foxp3 is a Potential Marker for CD4$^+$ Treg Cells in Patients with Rheumatoid Arthritis

Mengru Yang[a,b] Yan Liu[b] Biyao Mo[c] Youqiu Xue[b] Congxiu Ye[b] Yutong Jiang[a] Xuan Bi[a] Meng Liu[d] Yunting Wu[b] Julie Wang[e] Nancy Olsen[e] Yunfeng Pan[a] Song Guo Zheng[f]

[a]Division of Rheumatology, Department of Internal Medicine, the Third Affiliated Hospital, Sun Yat-Sen University, Guangzhou, China, [b]Center for Clinical Immunology, the Third Affiliated Hospital, Sun Yat-Sen University, Guangzhou, China, [c]Division of Rheumatology, Department of Internal Medicine, Hainan General Hospital, Haikou, China, [d]Division of Rheumatology, Department of Internal Medicine, Guangdong Second Provincial Central Hospital, Guangzhou, China, [e]Division of Rheumatology, Department of Medicine, Penn State University Hershey College of Medicine, Hershey, PA, USA, [f]Department of Internal Medicine, Ohio State University College of Medicine and Wexner Medical Center, Columbus, OH, USA

Supplemental figure 1

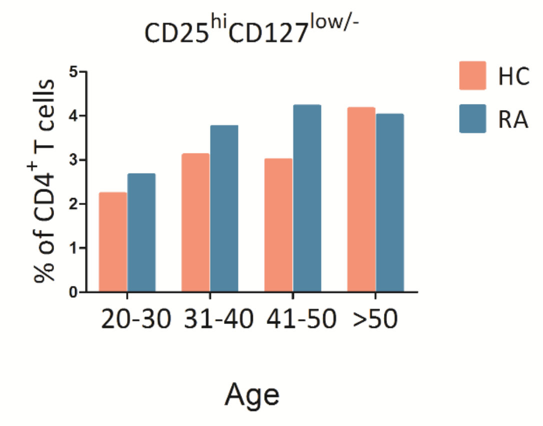

Supplemental figure 1. Frequencies of $CD4^{+}CD25^{hi}CD127^{low/-}$ Treg in peripheral blood of HC and RA patients of different ages.

Supplemental figure 2

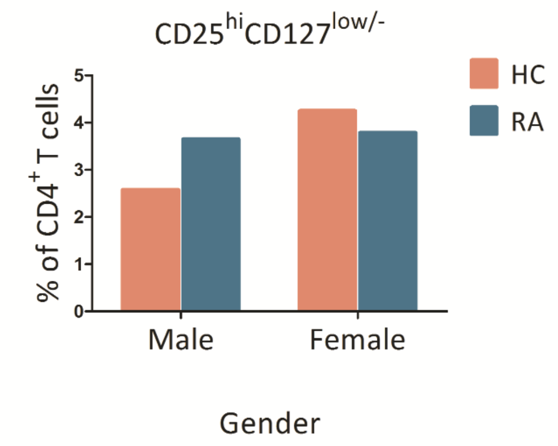

Supplemental figure 2. Frequencies of $CD4^{+}CD25^{hi}CD127^{low/-}$ Treg in peripheral blood of HC and RA patients of different gender.
